# Phylogeography of the neotropical *Anopheles triannulatus* complex (Diptera: Culicidae) supports deep structure and complex patterns

**DOI:** 10.1186/1756-3305-6-47

**Published:** 2013-02-22

**Authors:** Marta Moreno, Sara Bickersmith, Wesley Harlow, Jessica Hildebrandt, Sascha N McKeon, Teresa Fernandes Silva-do-Nascimento, Jose R Loaiza, Freddy Ruiz, Ricardo Lourenço-de-Oliveira, Maria AM Sallum, Eduardo S Bergo, Gary N Fritz, Richard C Wilkerson, Yvonne M Linton, Maria J Dantur Juri, Yadira Rangel, Marinete M Póvoa, Lina A Gutiérrez-Builes, Margarita M Correa, Jan E Conn

**Affiliations:** 1 New York State Department of Health, Wadsworth Center, Griffin Laboratory, Albany, NY, USA; 2 Department of Biomedical Sciences, School of Public Health, State University of New York, Albany, NY, USA; 3 Departamento de Entomología, Instituto Oswaldo Cruz-Fiocruz, Rio de Janeiro, Brazil; 4 Instituto de Investigaciones Científicas y Servicios de Alta Tecnología, Clayton, Panamá, República de Panamá; 5 Division of Entomology, Walter Reed Army Institute of Research, Silver Spring, MD, USA; 6 Departamento de Epidemiologia, Faculdade de Saúde Pública, Universidade de São Paulo, São Paulo, Brazil; 7 Superintendência de Controle de Endemias, SUCEN, São Paulo, Brazil; 8 Department of Biological Sciences, Eastern Illinois University, Charleston, IL, USA; 9 Instituto Superior de Entomología "Dr. Abraham Willink", Facultad de Ciencias, Naturales e Instituto Miguel Lillo, Universidad Nacional de Tucumán, Tucumán, Argentina; 10 Laboratorio de Biologia de Vectores, Instituto de Zoología y Ecología Tropical, Universidad Central de Venezuela, Caracas, Venezuela; 11 Instituto Evandro Chagas, Secção de psitologia, Belém, Brazil; 12 Grupo de Microbiología Molecular, Escuela de Microbiología, Universidad de Antioquia, Medellín, Colombia; 13 Present address: Division Infectious Diseases University of California San Diego, George Palade Labs, School of Medicine, 92093, 9500 Gilman Drive, MC 0741, La Jolla, CA, USA

**Keywords:** *Anopheles triannulatus* s.s., *Anopheles halophylus*, *Anopheles triannulatus* C, phylogeography, *COI* gene, *White* gene, ITS2

## Abstract

**Background:**

The molecular phylogenetic relationships and population structure of the species of the *Anopheles triannulatus* complex: *Anopheles triannulatus* s.s., *Anopheles halophylus* and the putative species *Anopheles triannulatus* C were investigated.

**Methods:**

The mitochondrial *COI* gene, the nuclear *white* gene and rDNA ITS2 of samples that include the known geographic distribution of these taxa were analyzed. Phylogenetic analyses were performed using Bayesian inference, Maximum parsimony and Maximum likelihood approaches.

**Results:**

Each data set analyzed septely yielded a different topology but none provided evidence for the seption of *An. halophylus* and *An. triannulatus* C, consistent with the hypothesis that the two are undergoing incipient speciation. The phylogenetic analyses of the *white* gene found three main clades, whereas the statistical parsimony network detected only a single metapopulation of *Anopheles triannulatus* s.l. Seven *COI* lineages were detected by phylogenetic and network analysis. In contrast, the network, but not the phylogenetic analyses, strongly supported three ITS2 groups. Combined data analyses provided the best resolution of the trees, with two major clades, Amazonian (clade I) and trans-Andean + Amazon Delta (clade II). Clade I consists of multiple subclades: *An. halophylus* + *An. triannulatus* C; trans-Andean Venezuela; central Amazonia + central Bolivia; Atlantic coastal lowland; and Amazon delta. Clade II includes three subclades: Panama; cis-Andean Colombia; and cis-Venezuela. The Amazon delta specimens are in both clades, likely indicating local sympatry. Spatial and molecular variance analyses detected nine groups, corroborating some of subclades obtained in the combined data analysis.

**Conclusion:**

Combination of the three molecular markers provided the best resolution for differentiation within *An. triannulatus* s.s. and *An. halophylus* and C. The latest two species seem to be very closely related and the analyses performed were not conclusive regarding species differentiation. Further studies including new molecular markers would be desirable to solve this species status question. Besides, results of the study indicate a trans-Andean origin for *An. triannulatus* s.l. The potential implications for malaria epidemiology remain to be investigated.

## Background

Neotropical anophelines have shown an extraordinary diversity and complexity due, in part, to the capability of dipterans to adapt to and utilize a broad variety of ecological niches [1]. The subgenus *Nyssorhynchus* provides an example of extensive morphological and genetic variation within taxa, and several studies have focused on the processes driving this differentiation. Some hypotheses are related to climatic changes that occurred in different epochs, provoking drastic modifications to the habitats of different organisms [2,3]. However, this remains controversial because of the paucity of available evidence-based data [4-6]. Furthermore, factors affecting speciation and population differentiation, such as ecology, behaviour and genetics, evolve at different rates and are not necessarily congruent [7].

Some of these taxa, estimated to be 10% of all anophelines, are directly responsible for malaria psite transmission and their accurate identification is necessary for the implementation of effective control strategies. Understanding the current distribution of species, investigating past or recent demographic events (population growth or contraction), gene flow, as well as human interventions (exploitation of new ecological niches, introduction of non endemic species, among others), can provide powerful tools and valuable predictors for the management of pathogens transmitted by anophelines.

*Anopheles triannulatus* s.l., subgenus *Nyssorhynchus*, [8] was first described from adult females in central Brazil and subsequently reported in Central America (Costa Rica, Nicaragua and Panama), in the majority of South American countries [9,10], and recently in some Caribbean islands [11]. This species has been previously described under different names (*syn.bachmanni* Petrocchi, *syn. chagasi* Galvao, *syn. cuyabensis* Neiva and Pinto, *syn. davisi* Paterson and Shannon, *syn. perezi* Shannon and Del Ponte). Morphological variation was later considered intraspecific and attributed to adaptation to different habitats [9,12-18]. However, recent investigations based on morphological characters of the male genitalia and immature stages led to the designation of a new species, *Anopheles halophylus,* and elevated the status from polymorphic species to complex, (i.e. the *Anopheles triannulatus* complex) [18-20].

Genetic distance analysis of allozymes and RAPD detected a third species, *An. triannulatus* C, and showed that *An. halophylus* and *An. triannulatus* species C formed a reciprocally monophyletic group [20]. Apart from this preliminary finding, very little is known about the phylogenetic relationships of members of the *An. triannulatus* complex, except for the results of analysis of sequences of *cpr* and *timeless* genes [21], which confirmed previous findings and suggested that *An. halophylus* and *An. triannulatus* C are in the process of incipient speciation.

Seasonal population density and behavioral differences have also been reported within the complex, for example, potential species-specific preferences for different larval habitats of species that occur in sympatry, e.g., *An. halophylus* and *An. triannulatus* species C [19,22].

*An. triannulatus* s.l. has been incriminated in human malaria transmission in different regions of Brazil [23-26]), and probably Peru and Venezuela [27,28], although the role of each species within the complex remains unknown. However, zoophilic and exophilic behaviour has been much commonly reported in Brazil [29-32].

The study of sibling species is aggravated by the difficulty of identification based exclusively on morphological characters when key traits, especially in adult females, may exhibit great phenotypic plasticity [33]. Nowadays, DNA sequences are an essential tool for delineating and identifying species, as well as for gathering information about the genetic variation within species complexes. The mitochondrial gene cytochrome oxidase subunit 1 (*COI*) is usually informative in phylogenetic reconstruction and geographic variability [34-36]. To represent the nuclear genome the single-copy *white* gene was chosen [37,38]. The ribosomal DNA (r-DNA) internal transcribed spacer 2 (ITS2) was used because it plays an important role in distinguishing cryptic anopheline species [39,40].

The major objective of this study was to reconstruct and clarify the evolutionary relationships based on mtDNA and nuclear sequences, to illustrate the demographic history at the population level, and to provide information on the distribution of *An. triannulatus* s.l. in several countries across its range. The information obtained would be useful to distinguish the species and help to focus scarce vector control resources on species involved in malaria transmission.

## Methods

### Sampling

**Figure 1:**
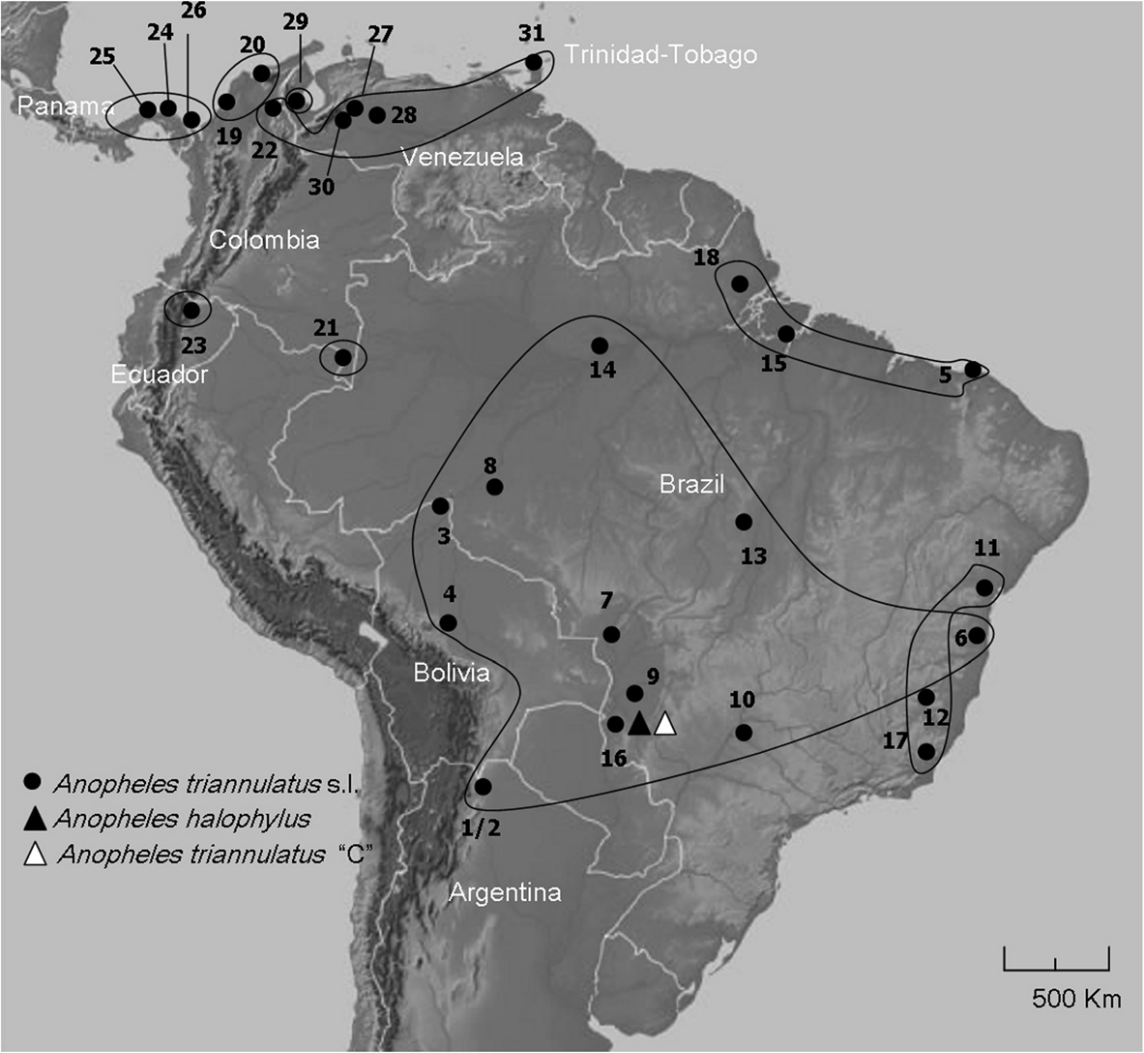
***Anopheles triannulatus *s.l. sampled localities.** The locality numbers correspond to those in Table 1. Black dots represent *Anopheles triannulatus* s.l., black and white triangles represent *An. halophylus* and *An. triannulatus* C type localities, respectively. The map depicts the *k* = 9 groups yielded by SAMOVA software and supported by AMOVA analysis.

Mosquitoes were collected from 31 localities in eight countries, including Argentina, Bolivia, Brazil, Colombia, Ecuador, Panama, Trinidad-Tobago and Venezuela (Table 1) covering a wide range of the reported species distribution (Figure 1). Adult mosquitoes were collected outdoors by human landing catches (protocols approved by the Institutional Review Board of the New York State Department of Health, University of Antioquia, University of Florida and University of Vermont), except specimens from Argentina and Brazil that were collected by CDC light traps or horse-baited Shannon traps, respectively. Some specimens included in the analysis from Brazil (donated by MAMS) were collected as either larvae or pupae and kept in the laboratory to obtain adults linked with larval and/or pupal exuviae. Two topotype specimens of *Anopheles halophylus* and *Anopheles triannulatus* C (Salobra, Brazil) and *An. triannulatus* s.s. were included in the analysis. Specimens were morphologically identified using the available identification keys [9,17]. Identification of *An. halophylus*, *An. triannulatus* C and some representatives of *An. triannulatus* s.s. was based on morphological characters and the *Mpi* diagnostic allozyme [20]. Mosquitoes were then stored in either 95% ethanol or on silica gel at room temperature until DNA extraction.

**Table 1:** **Summary data for collection localities and sample information of the *Anopheles triannulatus *complex**

**Site No.**	**Country**	**Locality**		***N***		**Coordinates**	**Collector**
			***COI *(*n*** **= 326)**	***white *(*n*** **= 85)**	**ITS2 (*n*** **= 34)**		
**1**	**Argentina**	Yuto Farm (FY)	23	3	1	23°63’S/64°46’W	MJD
**2**		Lake Yuto (LY)	3	0	0	23°38’S/64°27’W	MJD
**3**	**Bolivia**	Guayaramerin (GU)	4	0	0	10°49’S/65°21’W	JEC
**4**		Puerto Villaroel (PV)	10	2	2	16°52’S/64°46’W	JEC
**5**	**Brazil**	Ceara (CE)	2	2	0	5°05’S/40°23’W	TFS
**6**		Espirito Santo (ES)	1	0	0	1°15’N/50°54'W	TFS
**7**		Fazenda S. Joao (MT)	2	1	1	16°57’S/56°36’W	TFS
**8**		Monte Negro (RO)	5	2	0	10°15’S/63°19’W	MAMS/ESB
**9**		Aquidauana (Pantanal do Rio Negro) (AQU)	6	4	0	19°29’S/55°36’W	MAMS/ESB
**10**		Inubia Paulista (SP)	6	2	0	21°39’S/50°56’W	MAMS/ESB
**11**		Camacan/Santa Luzia (Bahia) (BA)	7	3	0	15°25’S/39°32’W	MAMS/ESB
**12**		Coronel Pacheco (Minas Gerais) (MG)	6	3	0	21°38’S/43°19’W	MAMS/ESB
**13**		Lagoa da Confusao (Tocantins) (TO)	6	2	0	10°35’S/49°41’W	MAMS/ESB
**14**		Itaituba (ITB)	5	1	1	4°15’S/55°59’W	MAMP/JEC
**15**		Oswaldo Cruz (OC)	19	7	3	8°00’S/35°00’W	MAMP/JEC
**16**		Salobra (MS)	20	9	4	20°12’S/56°29’W	TFS
**17**		Silva Jardim (RJ)	2	2	1	22°39’S/42°23’W	TFS
**18**		Tartarugalzinho (TAR)	23	4	1	1°30’N/50°54’W	MAMP/JEC
**19**	**Colombia**	Monitos (MO)	22	6	4	9°13’N/76°08’W	NN/PA
**20**		Santa Rosa de Lima (SO)	27	5	4	10°26’N/75°21 W	NN/JP
**21**		Leticia, km12 (COSW)	10	4	3	04°6'S/69°57'W	JFR
**22**		Tibu (COL)	3	0	0	08°38’N/72°44'W	JFR
**23**	**Ecuador**	Juan Montalvo (JM)	6	1	1	0°50’N/78°51’W	GNF
**24**	**Panama**	Bayano (BAY)	7	1	1	9°07’N/79°01’W	JRL
**25**		Gamboa (GAM)	29	4	2	9°07’N/79°42’W	JRL
**26**		Meteti (MET)	17	2	1	8°30’N/79°58’W	JRL
**27**	**Venezuela**	Boconoito (BOC)	6	2	0	8°50’N/69°58’W	JEC/YR
**28**		Cano Amarillo (CAM)	15	6	2	8°43’N/71°34’W	JEC/YR
**29**		Casigua Zulia (CAS)	25	4	2	8°44’N/72°30’W	JEC/YR
**30**		La Veguita (VG)	6	1	0	8°52’N/70°0’W	JEC/EB
**31**	**Trinidad-Tobago**	St.Andrew/St.David, Valencia (TRI)	2	0	0	10°39’N/61°09’W	RCW

Salobra (16) and Fazenda S. João (7), in Brazil, are the type localities for *An. halophylus* and *An. triannulatus* C, respectively. *N*, sample size of each of the genes sequenced; MJD, MJ Dantur; JEC, JE Conn; TFS, T Fernandes-da-Silva; MAMS, MA Mureb-Sallum; ESB, ES Bergo; MAMP: MAM Povoa; NN, N Naranjo; PA, P Aviles; JP, J Pinto; JFR, JF Ruiz; GNF, GN Fritz; JRL, JR Loaiza; YR: Y Rangel; EB: E Brown; RCW, RC Wilkerson.

### DNA extraction and gene amplification

Total genomic DNA was extracted from each specimen using the DNeasy tissue kit (Qiagen, CA, USA). Polymerase chain reaction was used to amplify a 1200 base pair (bp) fragment of the mtDNA *COI* gene using the primers UEA3 and UEA10 described in [41]. The *white* gene was also amplified via PCR using the primers WF and W2R [42,43]. The amplification of the ITS2 was performed following the protocol described in [44] with the primers 5.8S and 28S, yielding a 500 bp fragment. Each PCR reaction was carried out using a Ready-To-Go-PCR bead (Amersham Pharmacia/Biotech NJ, USA) and performed in a PTC-200 thermal cycler (BioRad Inc.). PCR products were purified on CentriSpin 40 columns (Princeton Septions, NJ, USA). Following standard PCR reactions, both strands were sequenced at the Applied Genomic Technologies Core (Wadsworth Center) on an ABI PRISM 3700 automated DNA sequencer.

### Sequence alignment and phylogenetic analysis

Sequencer 4.1 (Gene Codes Corps.) was used for automatic sequence alignment into contigs and proofreading sequences files; subsequently, all sequences were aligned with Clustal W and then compiled in Bioedit 4.0 [45].

The data sets defined for the subsequent analyses were: 1) the *COI* fragment, 2) the *COI* gene with nt1 + nt2 positions (to avoid redundancy in the nt3 position), 3) the *white* gene (with and without intron), 4) the ITS2 and 5) a combined concatenated data set of the three fragments. For each data set, an appropriate model of nucleotide substitutions was obtained with the software jModeltest 3.5 [46]. The models obtained were GTR + I + G for the *COI* gene (all positions), K81uf + I + G for *COI* gene (nt1 + nt2 positions), GTR + G for the *white* gene and HKY for the ITS2, selected by using the Akaike Information Criterion (AIC). These model pmeters were used as prior information in the subsequent maximum likelihood (ML) and Bayesian analyses.

To reconstruct phylogenetic relationships, four different approaches with each of the data sets were conducted. The ML analyses were performed using the heuristic search algorithm; both analyses were performed on the haplotypes with branch support evaluated by 1000 bootstrap replicates.

Bayesian inference (BI) analysis was performed using MrBayes 3.0.4 [47] allowing, in the combined data set, the specification of a distinct model and pmeters for the subset [48]. Different partitioning strategies were defined: P1) is a partition for each gene; P2) a partition for each gene without the 3^rd^ codon position for the mitochondrial marker and P3) the combined sequence without partitions. A Bayes factor (2lnB_F_) was used to choose the best partitioning strategies. Analyses were initiated with random starting trees and run for 10,000,000 to 20,000,000 generations, sampling every 1000 generations with a ‘burn-in’ of 25%. Posterior probabilities were used to assess nodal support. FigTree v1.2.1 was employed for visualization of the consensus trees.

### Population structure and genetic diversity analysis

Nucleotide and haplotype diversities were calculated within the main mitochondrial lineages, defined by the previous BI and the minimum spanning network, using DNASP v 5 [49].

A spatial analysis of molecular variance (SAMOVA) was performed to define groups of populations as geographically homogeneous and with a maximum of differentiation from each other, combining genetic and geographic sample information [50]. The random partition of the data was run by defining *K* = 2–20, with *K* number of groups to maximize the *F*_CT_ index, i.e., the proportion of total genetic variance due to differences between groups of populations [51].

**Figure 2:**
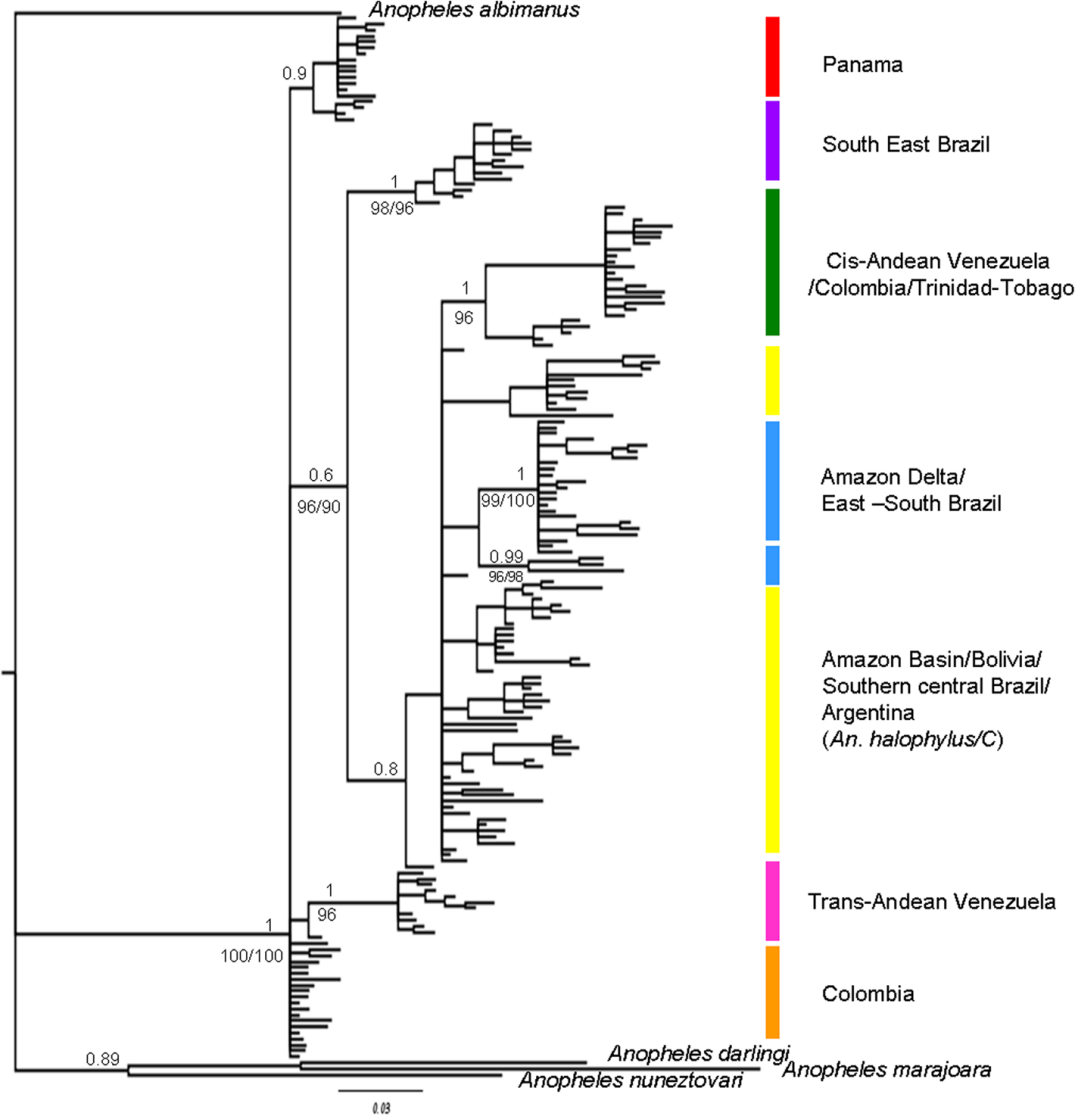
**Tree topology MP, ML and BI of the mitochondrial *COI *gene of the *Anopheles triannulatus *complex.** Both procedures produced trees of similar topology, with only branch support values above 70% shown. Bayesian posterior probability is above the branch and Maximum Likelihood bootstrapping (percentage) is below the branch. *Anopheles albimanus*, *Anopheles darlingi*, *Anopheles nuneztovari* and *Anopheles marajoara* were used to root the tree.

In addition, the characterization of patterns of genetic diversity variation in the *Anopheles triannulatus* Complex was performed with the analysis of molecular variance (AMOVA), with the option of establishing different hierarchical levels [51]. Groups of variation were defined based on SAMOVA, BI and networks (Figure 2): 1) 9 groups obtained from SAMOVA; 2) SAMOVA groups but with Ecuador (23; Figure 1) and Amazonian Colombia (21; Figure 1) together; 3) SAMOVA groups but combining Venezuela (29) with those from western Colombia (19, 20); and 4) SAMOVA groups but combining locality 29 with the Venezuelan and Trinidad-Tobago populations.

To describe patterns of population genetic variation that originated from spatially limited gene flow, isolation by distance (IBD) was tested with a nonpmetric Mantel test through the web-based computer program IBDWS v3.16 [52]. This analysis was applied only to populations in northwestern Latin America for which we have adequate regional (geographical) sampling. In this case, the distance was calculated as the straight line geographical distance between pairs of sampled localities using the Geographic Distance Matrix Generator tool [53] and the validation of the analysis was provided by 10,000 randomizations. Partial Mantel tests were performed to test for the effect of the Andes on genetic divergence [54].

### Phylogeographic analysis

To infer the haplotype relationships within the data sets, the median-joining network algorithm was performed [55], available in NETWORK v 4.5.1.0 (http://www.fluxus-engineering.com), which combines the topology of a minimum spanning tree with a parsimonious search for the missing haplotypes. To determine the ancestral network node, *COI* sequences of closely related species, *Anopheles nuneztovari* and *Anopheles darlingi* were included. Because this algorithm was designed for non-recombining molecules, a statistical parsimony network was used for the nuclear *white* gene and the ITS2 data [56] with a 95% confidence level, using the TCS v 1.21 software [57].

### Inference of population history

To investigate the historical demography of the *Anopheles triannulatus* Complex, neutrality tests were performed and applied to the SAMOVA groups and to the entire data set. The first two estimates (Tajima’s *D* and *R*_*2*_) are based on the frequency spectrum of mutation whereas Fu’s *F*s is based on linkage disequilibrium and haplotype distribution. To further confirm demographic expansion events, mismatch distribution analyses were conducted. The time of expansion was estimated using the formula *τ* = 2*ut*_e_, where *τ* is estimated in the mismatch distribution analysis, *u* is the nucleotide mutation rate in the specific DNA region and *t*_e_ is time since expansion. The mutation rate in the mitochondrial gene has been estimated from *Drosophila* as 1x10^-8^/site/year [58]. The time of the beginning of lineage divergence was calculated following *D*_A_/2 *k*_*,*_ where *D*_A_ is the average nucleotide divergence between lineages and 2 *k* is the divergence rate [59]. We utilized the *COI* gene substitution rate of 2.3% divergence per million years [60] and estimated these pmeters and their significance based on the coalescence process in DNASP v 5.10 [49] and Arlequin v3.11 [61].

## Results

A total of 326 specimens of the *Anopheles triannulatus* complex were sequenced for the mtDNA *COI* gene (Table 1). All sequences could be unambiguously aligned and no insertions or deletions were found. Translation into amino acid ruled out the presence of nuclear mitochondrial pseudogenes.

The final alignment of the *COI* gene (partial sequence) had a length of 689 bp, of which 202 characters were variable polymorphic sites and 154 were parsimony informative. For the single-copy nuclear *white* gene, 85 individuals randomly selected were included, representing each of the mtDNA-based lineages, and the *An. halophylus* and *An. triannulatus* C samples. A fragment of 646 bp with an intron of 70 bp was detected within the *white* gene sequences in all individuals, independent of the species. This intron was excluded for some of the analyses using a final fragment of 576 bp.

Finally, ITS2 was amplified from 34 individuals randomly chosen from the *COI* lineages, and the length varied from 542-570 bp (intra and interspecific differences), with a final alignment length of 483 bp (3’ and 5’ ends to make sequences equal length). All sequences were deposited in GenBank under Accession numbers *COI* (JN085964-JN086138), *white* gene (JF931140-JF931172) and ITS2 (JF972999-JF973009).

### Phylogenetic analysis

#### Mitochondrial DNA data

The Bayesian, ML and MP trees of the overall analysis are shown in Figure 2. Trees were rooted using Neotropical *Nyssorhynchus* species: *Anopheles darlingi, Anopheles marajoara, Anopheles nuneztovari* and *Anopheles albimanus* as outgroup [62]. The tree topologies obtained by the 50% majority rule consensus using BI, ML and MP in PAUP were basically in agreement, consistently recovering the *Anopheles triannulatus* complex as a monophyletic lineage. Two main clades were identified based on the geographic distribution of the samples (with some exceptions) and their genetic distances: one encompassing samples from Panama, northern Colombia and trans-Andean Venezuela, and the other (Amazonian) including samples from South America and cis-Andean Venezuela, and *An. halophylus* and *An. triannulatus* C. Within the Amazonian clade further subclades can be described according to tree topology and geographic origin of the samples (Figure 2). Both topologies suggest that the clade, which encompasses samples from Colombia is the most basal within the Complex. The main differences in the tree topologies were the recovery of Panama, Colombia and trans-Venezuela (northern) as a unique clade, although with low posterior probability branch support. The second clade (Figure 2) is shallower when estimated by MP, and not all the subclades are supported.

The samples identified as *An. halophylus* and *An. triannulatus* C were in a clade together with other individuals identified as *An. triannulatus* s.s. from southern Brazil, Bolivia and Argentina. In all tree topologies, *An. halophylus* shares the same haplotype as one individual from eastern Brazil originally identified morphologically and by *Mpi* as *An. triannulatus* s.s.

Analysis excluding the nt3 codon position was performed because of saturation issues [63], but this did not improve the resolution of the topologies. No evidence for saturation at any of the 3 positions was found using the test in [64] (Additional file 1).

#### Nuclear DNA data

**Figure 3:**
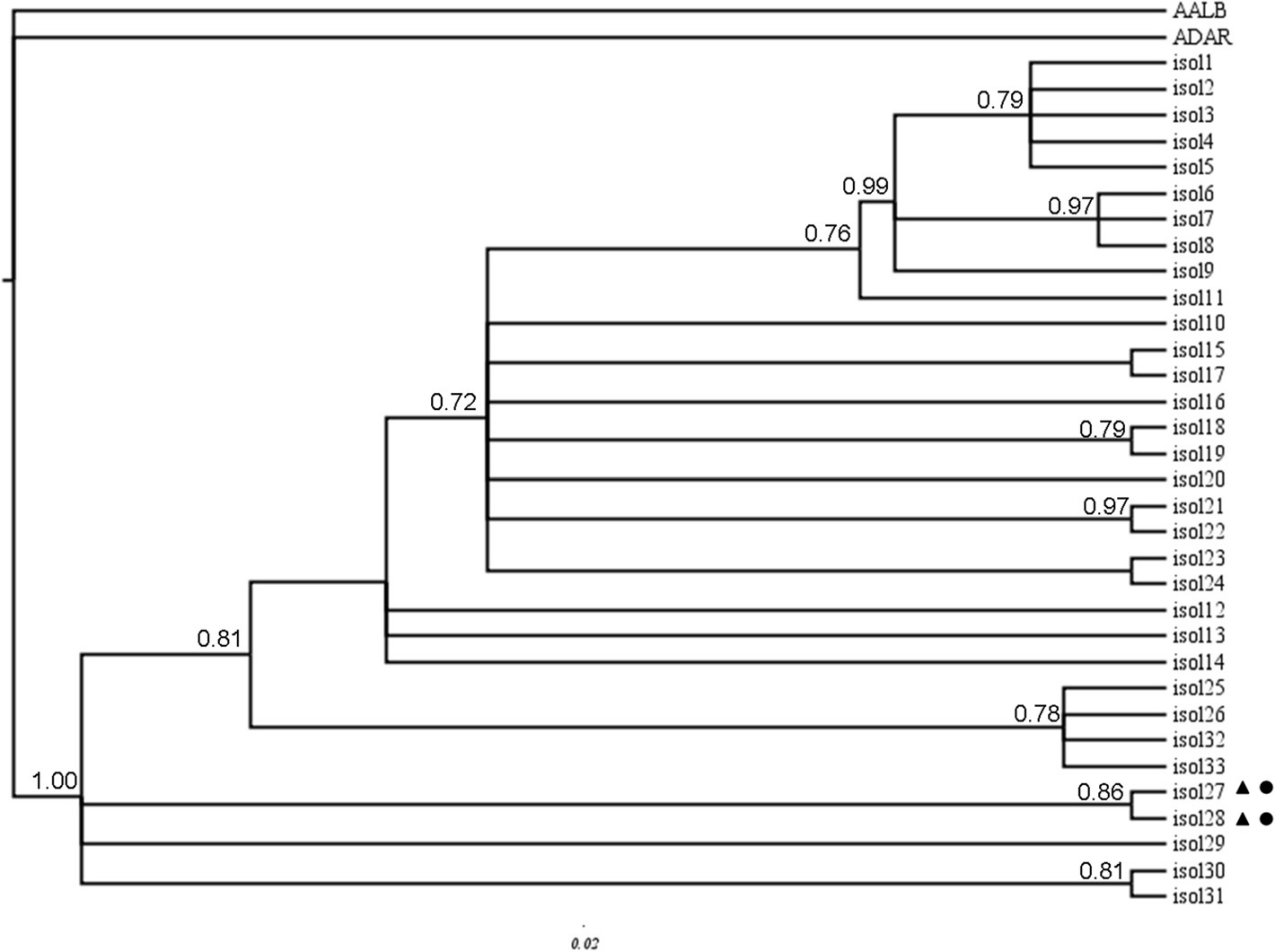
**Bayesian phylogenetic tree based on the *white gene *of the *Anopheles triannulatus *complex.** Black circles represent *An. triannulatus* C and black triangles *An. halophylus. Anopheles albimanus* (AALB) and *Anopheles darlingi* (ADAR) were used to root the tree.

A total of 33 haplotypes were identified among 85 individuals sequenced for the *white* gene. Bayesian tree (Figure 3) was similar to the three generated, employing MP analyses topology resolved a similar phylogeny, with some of the clades associated to geographic distribution. For instance, one major node includes individuals from central and northwestern South America and another includes samples from eastern and central Brazil (localities 5, 10, 13, Figure 1). In contrast, other individuals have low support or no geographic concordance, such as the genotypes from localities 4, 17 and 28. *An. halophylus* and *An. triannulatus* C clustered together with high support with two other *An. triannulatus* s.l. specimens from localities 1 and 7.

**Figure 4:**
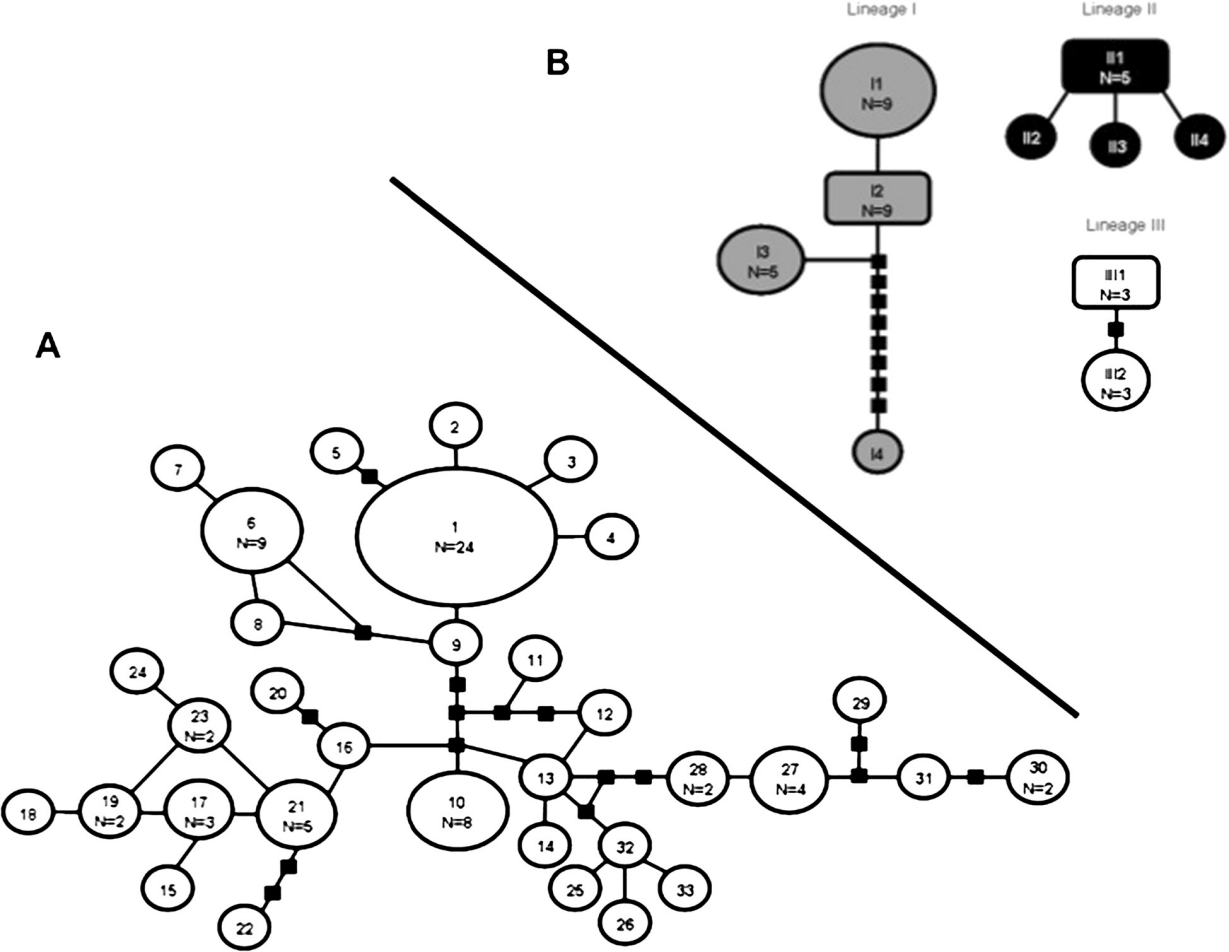
**Minimum spanning network of the 33 haplotypes of the *white *gene (A) and for the 11 haplotypes of ITS2 (B) from the *Anopheles triannulatus *complex (without intron).** The area of each circle is proportional to the frequency of the haplotype. Black squares represent missing or unsampled haplotypes and each segment connecting haplotypes represents one mutation.

All 33 unique *white* gene genotypes could be connected (Figure 4A). Genotype 1, with individuals from Panama, Colombia and Venezuela, was identified as ancestral [65]: the internal position in the network, the number of lineages that arise from it and the frequency (24 samples). The second most common genotype (number 6) is septed by only 3 mutational steps from genotype 1, and contains 9 samples from NE Brazil. Many of the remaining genotypes were unique (*n* = 23) and only eight were shared. The minimum spanning network depicted a complex pattern of relationships among the genotypes, and there was no support for the BI groups (Figure 2) in the mitochondrial or the nuclear gene genealogy.

Bayesian inference, maximum parsimony and maximum likelihood analysis of the ITS2 marker yielded similar topologies with only subtle differences in bootstrap and posterior probability support values (data not shown). The tree topologies showed some possibly basal individuals from diverse localities (Colombia, Panama, Brazil, and Venezuela) and two fairly well supported subclades: one of them recovers *An. halophylus* and *An. triannulatus* C as a single monophyletic clade. The other clade contains individuals from central Brazil, southern Colombia, Ecuador and Bolivia. In addition, the ITS2 network (Figure 4B) clearly depicts 3 main groups in the *Anopheles triannulatus* Complex; lineage I recovered individuals from Panama, trans-Andean Venezuela, NE Brazil and SE Brazil whereas lineage II comprised Amazon Basin and trans-Andean Venezuela individuals. Lineage III consisted of two genotypes septed by 1 mutation step with the *An. halophylus* and *An. triannulatus* C specimens, plus two individuals from Brazil and Argentina (originally identified as *An. triannulatus* s.l.), respectively. The deep differentiation of populations septed by the Andes, detected by the *COI* data, is also strongly supported with ITS2 lineages I and II.

#### Combined mitochondrial and nuclear DNA data

**Figure 5:**
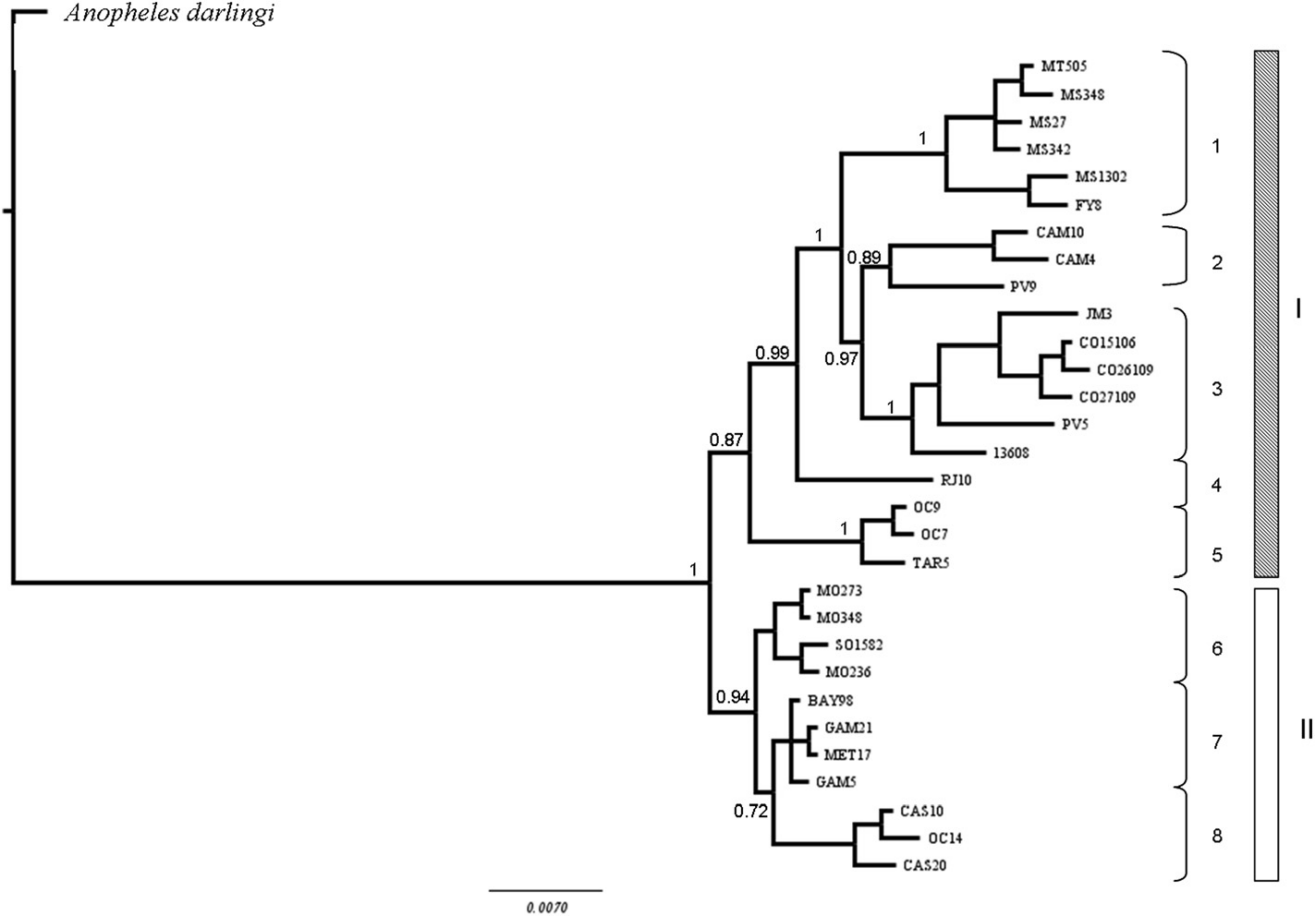
**Bayesian analysis of the concatenated *COI*** + ***white *gene + ITS2 alignment.** Support values for common branches are represented above branches estimated using a partition for each gene: GTR + I + G for the *COI* gene (all positions), GTR + G for the *white* gene and HKY for the ITS2 model of evolution. The outgroup *Anopheles darlingi* was used to root the tree.

Analysis of partitioning strategies showed the P1 model (independent evolutionary models for each gene) as the most accurate [66]. *B*_*F*_-based statistics also indicated this partition as the better fit, and the tree derived from these analyses is shown in Figure 5. In general, the combined data presented high posterior probability values. The tree topology recovered two main clades; clade I consists of multiple subclades: (1) *An. halophylus* + *An. triannulatus* C; (2) trans-Andean Venezuela; (3) central Amazonia + central Bolivia; (4) Atlantic coastal forest; and (5) Amazon delta. Clade II includes three subclades: (1) Panama; (2) cis-Andean Colombia; and cis-Venezuela. The Amazon delta specimens are in both clades (OC9, OC7 in Clade I and OC14 in Clade II), perhaps indicating sympatry of discrete taxa. *Anopheles halophylus* and *An. triannulatus* C were recovered as a single well-supported monophyletic subclade (1) together with some *An. triannulatus* s.l. individuals from the same type locality (Salobra) and from nearby Mato Grosso and Argentina. The other subclades (2–5) generally had a geographic component except that samples from Bolivia are found in both subclades 2 (PV9) and 3 (PV5) (Figure 5).

### Population structure and genetic diversity analysis

**Figure 6:**
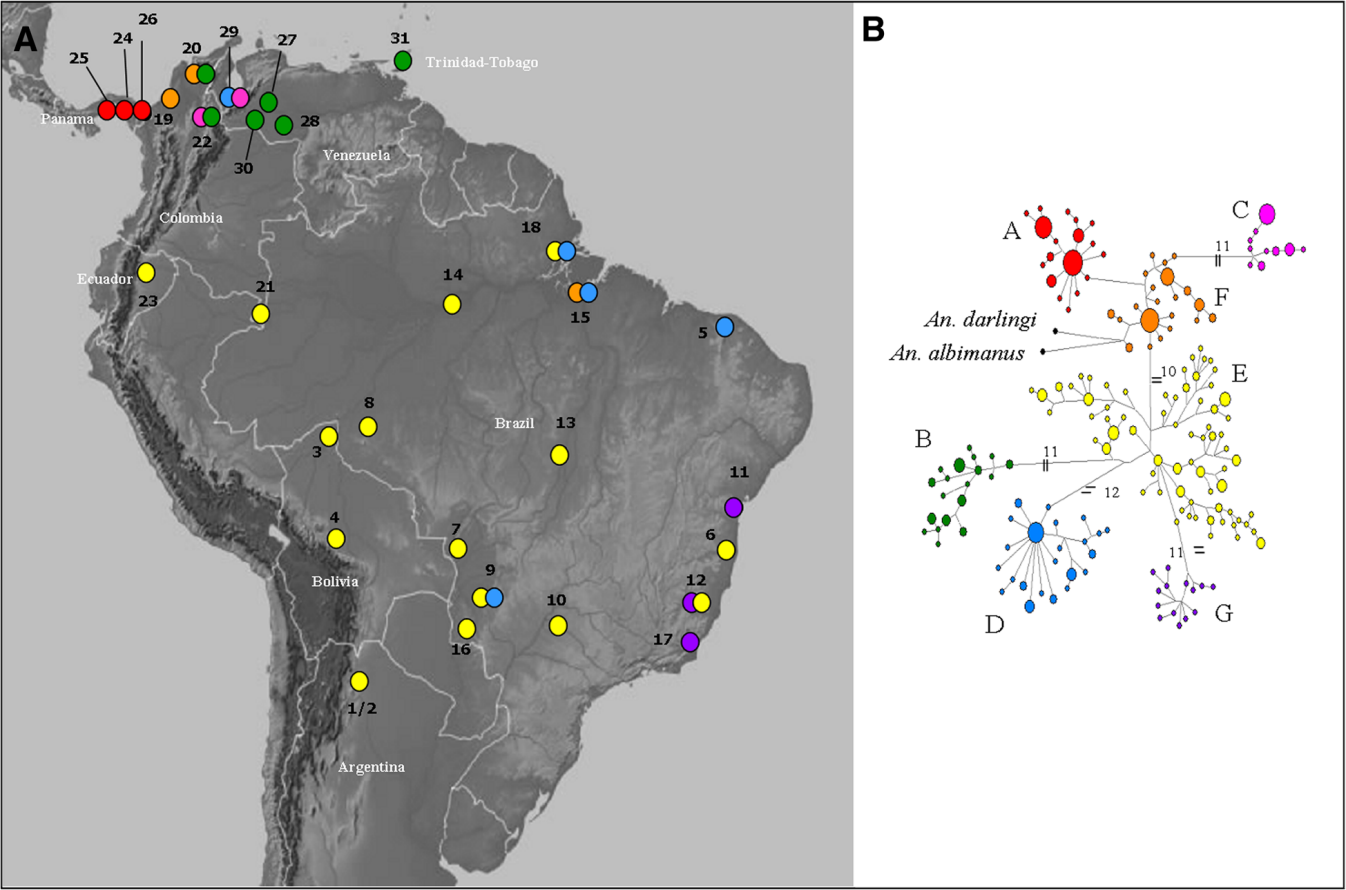
**Haplotype network derived from 175 haplotypes from 689 bp of the *COI *gene sequences from 326 specimens of the *Anopheles triannulatus *complex. A**) Geographic distribution of the *COI* lineages; **B**) Haplotypes are represented by circles and their frequency is proportional to the area. Numbers represent mutational steps between haplotypes and grey dots are median vectors. The *An. darlingi* and *An. nuneztovari* outgroup sequences (black dots) joined the network at the Colombian haplotypes and they are septed from all the *Anopheles triannulatus* complex individuals by more than 50 fixed differences.

The 326 sequences of the *COI* gene yielded one hundred and seventy-five haplotypes, with some shared between species, for instance, for *An. triannulatus* C and *An. triannulatus* s.s (see Table 2). The haplotype network reveals an intricate topology (Figure 6): from Panama, haplotypes A4 (*n* = 12) and A9 (*n* = 16), were the most common; C6 (Venezuela and Brazil) and D12 (NE Brazil) each comprised 11 individuals, whereas F11 (NW Colombia) consisted of 15 individuals. *An. halophylus* (E8 and E53) present haplotypes intermingled with *An. triannulatus* s.s. from eastern Brazil and Mato Grosso. Conversely, both *An. triannulatus* C haplotypes (E7 and E9) were unique.

**Table 2:** **Description of shared *COI *haplotypes for the *An. triannulatus *complex**

**Site no.**	**Country**	**Locality**	***N***	**Haplotypes**	**Lineages**
**1**	**Argentina**	Yuto Farm (FY)	23	**E1**,E2,E4,E15,**E44**(2),E45,E46,E47,**E50**(2),E51,**E52**,E57, E66(3),**E67**(4),E68,E69	E
**2**		Lake Yuto (LY)	3	**E58**,**E67**,E70	E
**3**	**Bolivia**	Guayaramerin (GU)	4	E32(4)	E
**4**		Puerto Villaroel (PV)	10	E3,E5,E6,E12(2),E13,E49,E62,E63(2)	E
**5**	**Brazil**	Ceara (CE)	2	D21,D22	D
**6**		Espirito Santo (ES)	1	**E53**	E
**7**		Fazenda S. Joao (MT)	2	E61,**E8**	E
**8**		Monte Negro (RO)	5	E22, E23,**E59**(2),**E65**	E
**9**		Aquidauana (Pantanal do Rio Negro) (AQU)	6	D23,E17,E18,E19,E20,E21	D,E
**10**		Inubia Paulista (SP)	6	**E1**,E28,E29,E30,**E48**,**E58**	E
**11**		Camacan/Santa Luzia (Bahia) (BA)	7	G3,G4,G5,G6,G7,G8,G9	G
**12**		Coronel Pacheco (Minas Gerais) (MG)	6	G10,G11,G12,G13,G14,E31	G,E
**13**		Lagoa da Confusao (Tocantins) (TO)	6	E24, E25, E26, E27,**E56,E65**	E
**14**		Itaituba (ITB)	5	E16,**E50**,**E56**,E64,**E65**	E
**15**		Oswaldo Cruz (OC)	19	**D1**,D5,D6,D7,D8,**D12**(7), D13(2),D14(2),D15, D16,**C6**	C,D
**16**		Salobra (MS)	20	**E1**,E7,**E8**(2),E9,E10,E11,E14(2),**E44**,**E48**, **E52**(4),**E53**,E54,E55,**E59**,E60	E
**17**		Silva Jardim (RJ)	2	G1,G2	G
**18**		Tartarugalzinho (TAR)	23	D2,D3,D4,D9,D10(4),D11,**D12**(4),D17,D18(4), D19(2),D20, E43(2)	D, E
**19**	**Colombia**	Monitos (MO)	23	**B3**,**F1**,F2, F3, **F4**(2),**F8**,**F9**, **F11**(9),F12(2),F13,F14,F16,F17	B,F
**20**		Santa Rosa de Lima (SO)	27	B18, **F1**,**F4**(6),F5(2),F6,F7,**F8**,**F9**(3), F10,**F11**(6),F15, F18,F19,F20	B,F
**21**		Leticia, km12 (COSW)	10	E36,E37(4),E38,E39,E40,E41,E42	E
**22**		Tibu (COL)	3	**B14**,B19,C11	B,C
**23**	**Ecuador**	Juan Montalvo (JM)	6	E33(4), E34, E35	E
**24**	**Panama**	Bayano (BAY)	7	**A3**,**A4**,**A9**,A14, **A16**(2), A18	A
**25**		Gamboa (GAM)	29	A1,A2,**A3**,**A4**(3),A5,A6,A7,A8,**A9**(10),A10 (4) A11,A12,A13,**A16**,A17	A
**26**		Meteti (MET)	17	**A4**(8),**A9**(5), A14,A15,**A16**(2), A19	A
**27**	**Venezuela**	Boconoito (BOC)	6	B1, **B2**, **B3**, B8(2),**B9**	B
**28**		Cano Amarillo (CAM)	15	**B2**,**B3**, B4, B5, **B9**,B10(2), B11, B12(2), B13, **B14**(4)	B
**29**		Casigua Zulia (CAS)	25	**D1**,C1(4),C2,C3(2),C4,C5, **C6**(10),C7,C8,C9(2),C10	D,C
**30**		La Veguita (VG)	6	B6,**B7**,B15,B16,B17,B18	B
**31**	**Trinidad-Tobago**	St.Andrew/St.David, Valencia (TRI)	2	**B7 (2)**	B

The number in parentheses indicates the frequency of the haplotype at that site; bold numbers are shared haplotypes, plain numbers are unique haplotypes in that population and underlined numbers are *An. halophylus* (E8, E53) and *An. triannulatus* C (E7, E9).

Overall, the *Anopheles triannulatus* complex network consists of 7 lineages, very similar to the BI, and depicts some relationships between haplotypes and geographic distribution, which may indicate variation in the population histories (Figure 6). Star shaped lineage A, suggestive of population expansion, is restricted to Panama. Other geographically nearby lineages, septed only by 2 mutational steps, are formed by trans-Andean Colombian (F); but there are >11 mutation steps between A and the western Venezuelan samples (C). In contrast, lineage B, consisting of haplotypes from cis-Andean Venezuela, Colombia, and Trinidad and Tobago, is > 21 mutational steps distant. Lineage E is of mixed origin: samples from Ecuador, Bolivia, Amazonian Colombia, southern Brazil, Argentina and a few haplotypes from central and eastern Brazil, mostly as singletons. Lineage D is mainly composed of eastern Brazilian populations with a star shape suggesting an expansion. Lineage G is restricted to populations from SE Brazil, east of the Central Mountain Range.

The SAMOVA *F*_CT_ values increased with the number of groups. However, the graphical representation of *F*_CT_ showed that at *k* = 9, a plateau was reached (*F*_CT_ = 0.53778) concordant with the most statistically significant AMOVA groups (Table 3).

**Table 3:** **Analysis of Molecular Variance (AMOVA) using *COI *in the *An. triannulatus *complex**

**Groups (localities)**	**Among groups**	**Among populations within groups**	**Within populations**
**1)**	53.81	3.24	42.95
**I:** 24, 25, 26	Φ_CT_ =0.53***	Φ_ST_ =0.57***	Φ_SC_ =0.07***
**II:** 19,20			
**III;** 5, 15, 18			
**IV:** 29			
**V:** 22, 27, 28, 30, 31			
**VI:** 23			
**VII:** 11, 12, 17			
**VIII:** 21			
**IX:** 1,2,3,4,6,7,8,9,10,13,14,16			
**2)**	53.13	3.90	42.96
**VI:** 21,23	Φ_CT_ =0.531***	Φ_ST_ =0.57***	Φ_SC_ =0.08***
**3)**	47.11	10.45	42.44
**II:** 19,20,29	Φ_CT_ =0.47***	Φ_ST_ =0.57***	Φ_SC_ =0.19***
**4)**	37.49	19.18	43.33
**IV:** 22, 27, 28, 29, 30, 31	Φ_CT_ =0.37***	Φ_ST_ =0.56***	Φ_SC_ =0.3***

1) Groups (localities number) derived from SAMOVA k = 9; 2), 3) and 4) with modifications in the groups based on SAMOVA results. ***p<0.001.

A Mantel test showed a significant correlation of pairwise genetic differences between the Panamanian, Colombian and Venezuelan samples with their respective geographic distances (*r* = 0.6564, *p* < 0.001), suggesting that distance accounts for approximately 65% of the genetic differentiation for this region. Furthermore, a partial Mantel test was performed to test the Andes as a barrier to gene flow between mosquito populations from Panama (trans) and Colombia and Venezuela (cis). In this case, the distance still added significant effect to the correlation when the Andean range was primarily considered (*r* = 0.6463; *p* < 0.01) (controlling for geography: *r* = −0.0115; *p* = 0.5860).

### Inference of *COI* population history

**Figure 7:**
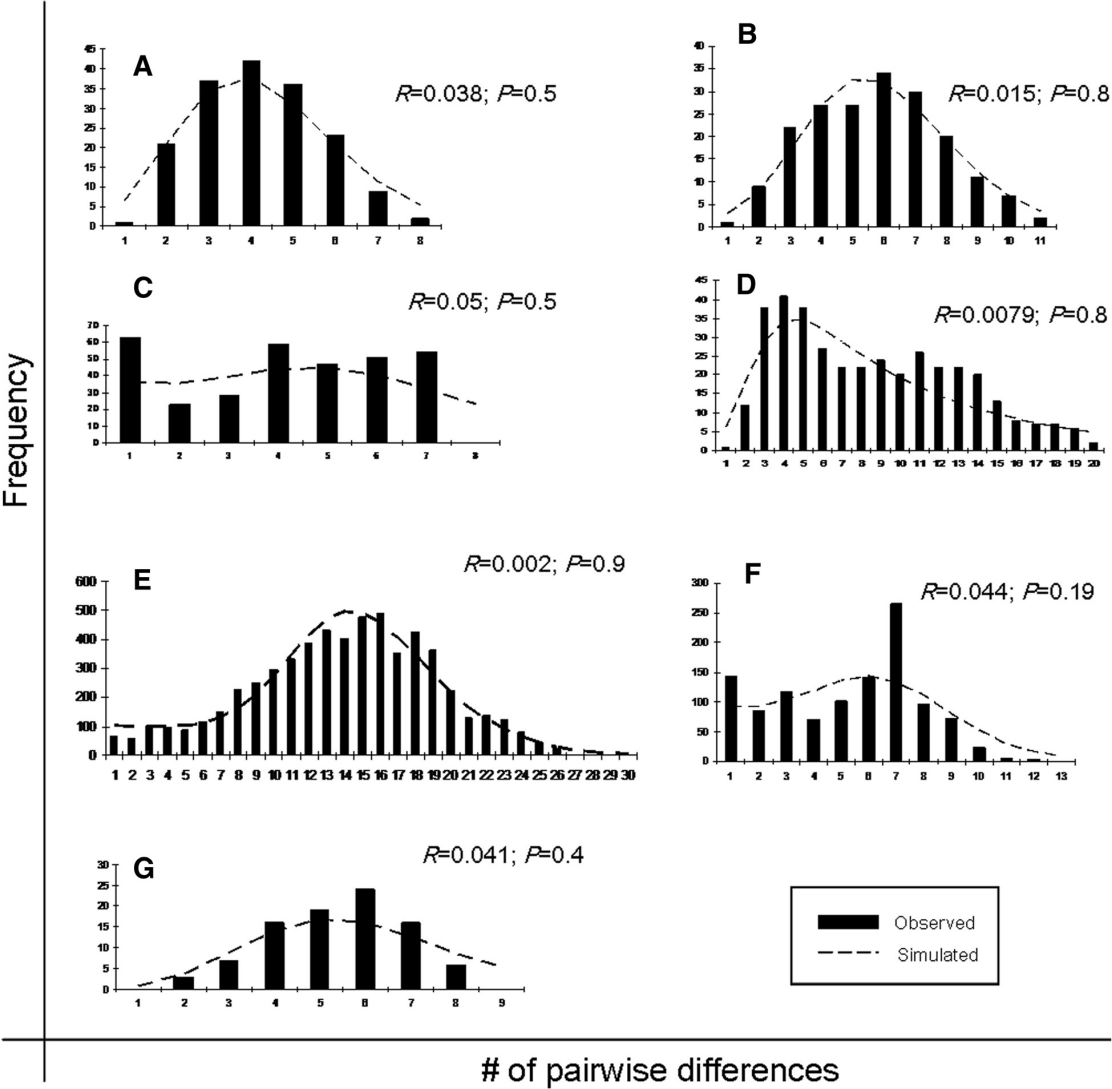
**Observed and simulated mismatch frequency distributions under population expansion model for each major *COI *mtDNA lineages of the *Anopheles triannulatus *complex.** Curves represent the frequency distribution of pairwise differences and *p*-values represent the probability that the variance of the simulated data set is equal to or greater than the observed data set.

All the neutrality tests in lineage A were significant, strongly indicating a demographic expansion (Table 4). All the other lineages presented significant *Fs* and *R*_*2*_ statistics. In addition, the unimodal mismatch distribution and the nonsignificant raggedness statistics are congruent with a model of sudden expansion in all lineages (Figure 7). A range of early to late Pleistocene divergence was identified in all the lineages, with the oldest estimation for widespread lineage E. However, visually, lineage E mismatch distribution and the haplotype network are more consistent with an older lineage close to equilibrium. Interestingly, if we combined Venezuelan lineages (B and C, excluding the single D1 haplotype), they depicted two different peaks in the mismatch distribution, corresponding to cis- and trans-Andean populations (395,821-327,737 years ago, respectively). The IBD test may be showing positive correlation with the distance as an artifact, because of the demographic expansion of the populations included in the analysis.

**Table 4:** **Molecular diversity and neutrality test in the main mt-DNA lineages within the *Anopheles triannulatus *complex**

***COI *gene lineage**	***N***	***h***	***π***	***Hd *(SD)**	***k***	***Fs***	F*	D*	***D***_***T***_	***R***^***2***^
A	53	18	0.00275	0.841 (0.035)	1.894	−11.706***	−3.223**	−3.205*	−1.802*	0.162***
B	33	19	0.00776	0.960 (0.018)	5.348	−8.354**	−1.492	−1.29	−1.21	0.160***
C	26	11	0.00457	0.806 (0.071)	3.148	−2.558	0.106	−0.0009	0.305	0.126***
D	43	23	0.00628	0.921 (0.030)	4.328	−11.479***	−1.373	−0.981	−1.483	0.109***
E	109	70	0.0194	0.989 (0.003)	13.383	−41.832***	−1.972	−1.999	−1.173	0.090***
F	48	20	0.00760	0.906 (0.029)	5.238	−6.361*	−2.775*	−2.748*	−1.575	0.109***
G	14	14	0.00636	1 (0.027)	4.385	−11.622***	−1.588	−1.429	−1.265	0.151***
*All*	326	175	0.0242	0.989 (0.0017)	16.676	−158.48***	−1.924	−2.334*	−0.914	0.075***

A: Panama; B: cis-Andean Venezuela + Colombia + Trinidad-Tobago; C: Venezuela; D: NE and C Brazil + Venezuela; E: Ecuador, Colombia, Bolivia, Argentina, Brazil; F: Colombia; G: SE Brazil; *N*: sample size; *h*: number of haplotypes; *π*: nucleotide diversity; *Hd*: Haplotype diversity (SD: Standard deviation); *k:* average number of pairwise differences; *F*_*S*_: Fu’s *F*_*S*_ statistic; F*: Fu and Li *F* test; D*:

Fu and Li’s *D* test; *D*_*T*_: Tajima’s *D*; *R*^*2*^: Ramos-Onsins & Rozas *R*^*2*^ test. *p < 0.05; **p < 0.02; ***p < 0.001.

A McDonald-Kreitman (MK) test was performed for each lineage to detect departures from neutral theory at the molecular level. There was no evidence of selection on either *COI* or *white* genes in several locations tested, using different species of *Anopheles* as outgroups.

## Discussion

### Taxonomic status of the *Anopheles triannulatus* complex

This study assesed the phylogenetic relationship of the *An. triannulatus* complex based on mitochondrial and nuclear data. Results of phylogenetic analysis showed that *An. halophylus* and *An. triannulatus* C do not form septe monophyletic clades, indicating that they are probably very closely related or are incipient species as proposed by Silva-do-Nascimento and collaborators [20,21]. The *COI* uncorrected pairwise genetic distances detected a 0.2% divergence between these putative taxa, whereas with those identified as *An. triannulatus* s.s. the range varied from 1.7-2.3%, depending on the sample. Therefore, this study supports the conclusion that *An. halophylus* and *An. triannulatus* C are more closely related to each other than either is to *An. triannulatus* s.s. [20,21]. Furthermore, the combined three gene analysis recovered a highly supported single clade consisting of *An. halophylus* and *An. triannulatus* C, whereas the mitochondrial *COI* gene provided a tree with lower resolution (short branches, low support) for the three species.

Diverse processes in the mitochondrial marker such as introgression or incomplete lineage sorting may be responsible for the inconclusive phylogenetic analysis within the complex. In addition, representatives from Argentina and Mato Grosso (Brazil) were recovered in every tree topology clustered together with *An. halophylus* and *An. triannulatus* C, and may represent a more extensive distribution of this taxa. Actually, specimens morphologically similar to *An. halophylus* and *An. triannulatus* C were reported in Western Brazilian Amazon, pguay and Bolivia [15,19,22,29,67].

Evidence of shared haplotypes, i.e. between *An. halophylus* and *An. triannulatus* s.s. and between *An. triannulatus* s.s. and *An. triannulatus* C were also found with this gene. The presence or absence of the intron in the *white* gene is an evolutionary event that appears more than once in the history of anophelines [68,69]. Within the *An. triannulatus* complex, all three species retained this intron and there were no significant nucleotide differences among their sequences.

The divergence of ITS2 lineages was very low within (0.2% in lineage I and II and 0 in lineage III) and between lineages (0.7% between I and II; 1% and 1.1% between I and III and II and III, respectively). Therefore, the ITS2 findings alone did not conclusively differentiate *An. halophylus* and *An. triannulatus* C, which suggest that a low level of gene flow may occur or have taken place in the recent past, as it has been proposed when analyzing sympatric specimens from Brazil with sequences of *timeless* gene alone [21]. On the other hand, the analysis of *cpr* gene sequences of the same specimens revealed fixed differences and considerable genetic differentiation between *An. halophylus* and *An. triannulatus* C [21].

The phylogenetic reconstruction supports monophyly of the *An. triannulatus* complex. The three genes detected different phylogenetic relationships as well as phylogeographic patterns in this complex. Nuclear DNA markers are expected to provide older demographic information than mtDNA because dissimilar effective population size can affect estimations of coalescent time [70]. Therefore, the genetic relationships that we obtained with the *white* gene support an original metapopulation, across the entire known geographic distribution, with posterior divergence in some populations, reflected in the results of the *COI* gene. The mitochondrial loci in most species have a shorter expected coalescence time compared with nuclear loci (only one-fourth of the effective population size), and thus there is a greater probability that the mitochondrial gene tree will accurately reflect the species tree [71].

On the other hand, the ITS2 marker did not show different lengths between putative species, but see [44]. The characteristic mutation replacement of this locus (elevated number of indels and low frequency of replacement mutations) may be more useful for species-specific PCR diagnostics in species complex members [72,73] than for phylogenetic inference.

The *COI* fragment was useful to describe the genetic structure of the *An. triannulatus* complex. However, the genetic variation and divergence within and between lineages might reach a different conclusion if this study had been based on the more conservative “Folmer region” (DNA barcode standard) [74].

### Divergence and demographic expansion of the *Anopheles triannulatus* complex

*Anopheles triannulatus* s.l. contains cryptic and geographically distinct mtDNA lineages, indicative of either speciation or substantial isolation and divergence among populations. The most common process of mosquito speciation has been considered to be the allopatric mode ([72] and references therein). Although the Amazon delta has been hypothesized as a natural barrier for populations of *An. triannulatus* and *An. darlingi*[5,43], in the current study populations of *An. triannulatus* located on both shores were part of a single lineage with shared haplotypes for both the mitochondrial and the nuclear markers. We performed AMOVA analysis with our data based on the recent findings of Pedro and collaborators [5] to test Amazonian groups of *An. triannulatus*. The five groups suggested in the cited study only contributed 21.64% to the variance, whereas our 3 groupings (TAR + CE + OC; MG + RJ + BA; and the remaining Amazonian populations yielded a significant variance of 41.27% (*p* < 0.01).

The high differentiation among populations in the Caribbean Andes region (Colombia-Venezuela, cis-trans Andean) suggests that the Sierra Nevada (west of Lake Maracaibo, Venezuela) acts as a partial barrier to gene flow for *COI* and *white* gene, and as a complete barrier for ITS2. The Andean orogeny seems to be responsible for this large source of variation, promoting isolation and secondary contact among different lineages in several taxa such as butterflies, birds, bees, sandflies [75], including neotropical anophelines such as *An. darlingi*[3], *An. albimanus*[76] and *An. nuneztovari*[43]. Furthermore, the bimodal *COI* mismatch distribution for all Venezuelan populations supports the hypothesis of two independent migration events [77]. The current study identified the Serranía de Perija in the Eastern Andes cordillera (between Venezuela and Colombia) as a porous genetic barrier to *An. triannulatus*, with some mtDNA haplotypes shared as well as some *white* gene exchange between cis-trans Andean populations. The ITS2 marker depicted the eastern Sierra Nevada Venezuelan populations as more closely related to those from Brazil, Ecuador and Bolivia.

Taking into account lineage divergence estimates in this study, climatic changes during the Pleistocene could have influenced this isolation, creating refugia on both sides of the mountains [2], and might explain better the lineage distributions than vicariant events associated with the older uplift of the North Western Andes (from Late Cretaceous to Holocene) [78]. The absence of shared *COI* haplotypes may indicate lack of gene flow between some of the lineages. Although the Mantel test showed a positive significant IBD pattern in this area, the results may be confounded by the recent expansion events [79].

In southern South America, the Coastal Mountain Range in SE Brazil promotes population structure in *An. darlingi*[80], and incipient speciation in *Anopheles cruzii*[81]. In fact, an extra-Andean glaciation event led to a Pleistocene refugium in Mount Itatiaia and the Serra do Mar [82]. Restricted gene flow and genetic structure of *An. triannulatus COI* lineage G in this region support a common phylogeographic pattern in mosquitoes and in some other similarly distributed species, such as sandflies [83]. An alternative explanation is that the most extensive *An. triannulatus* lineage E (Figure 6) could be interpreted as evidence of a Pleistocene range expansion combined with genetic exchange.

Although the origin of the *An. triannulatus* complex has been hypothesized to be south of the Amazon River [5], probably in the Belém or Tapajós refugia [84], our data provide mixed signals. On one hand, both *COI* and *white* gene coalescent and phylogenetic analyses found the most likely ancestral sequences to be in northwestern South America (Colombia, Panama, and/or Venezuela). On the other hand, the lowest nucleotide diversity values were found in these populations, and higher values in localities from the Amazon Basin. The low π value in Panamanian locations has been hypothesized in the malaria vector *An. albimanus* to be the result of a past bottleneck event followed by a demographic expansion dated approximately ~22,000 years ago [85], although recent bottlenecks can also mimic the effects of an expansion [86]. Panamanian populations of *An. triannulatus* included in the present study seem to have a similar demographic history and estimation dates for the proposed expansion (~ 23,000 years ago). Besides, the least polymorphic population may reflect a smaller effective size and relative isolation of a population. However, populations of *An. triannulatus* from the Amazon Basin seem to be in equilibrium, whereas in those from the Amazon Delta a demographic expansion around 15,043 years ago was detected (4265–99,387, 95% CI).

## Conclusions

Many malaria control programs focus on local vector management and the incrimination of species involved in malaria transmission is crucial. Molecular taxonomy can accurately identify malaria vectors [87]. Phylogeography has been informative in assessing historic migration and colonization routes for *An. albimanus*[88] and can also be used to trace the origin of accidental introductions such as *Aedes albopictus*[89]. Because *An. triannulatus* has been reportedly incriminated in malaria transmission in eastern Amazonian Peru and Amazonian Brazil and Venezuela [24,26,27,90] we hypothesize that at least lineage E is likely a malaria vector.

## Competing interests

The authors declare they have no competing interests.

## Authors’ contributions

MM, SB, WH, JH and SNM participated in the design of the study, performed molecular and genetic analysis and wrote the manuscript. TFSN, JRL, FR, RLO, MAMS, ESB, GNF, RW, YML, MJDJ, YR, MMP, LAG, MMC provided samples used in this study, advised on the analysis and assisted and helped to draft the manuscript. JEC conceived and supervised the study, and assisted in the writing of the manuscript. All authors read and approved the final manuscript.

## Supplementary Material

Additional file 1 **Numbers of transitions (X and s) and transversions (Δ and v) at each codon position plotted against Tamura-Nei (Tamura and Nei, 1993) genetic distances for the mitochondrial *COI *gene.** The steeper slope of the transitions in nt3 suggests no substitution saturation and is evidence for a constant rate of evolution. Click here for file
