# Supplementary figures and images for: Phylogeography of the neotropical Anopheles triannulatus complex (Diptera: Culicidae) supports deep structure and complex patterns
Source: Parasit Vectors. 2013 Feb 22;6:47. doi: 10.1186/1756-3305-6-47 (PMC3606328; doi:10.1186/1756-3305-6-47)

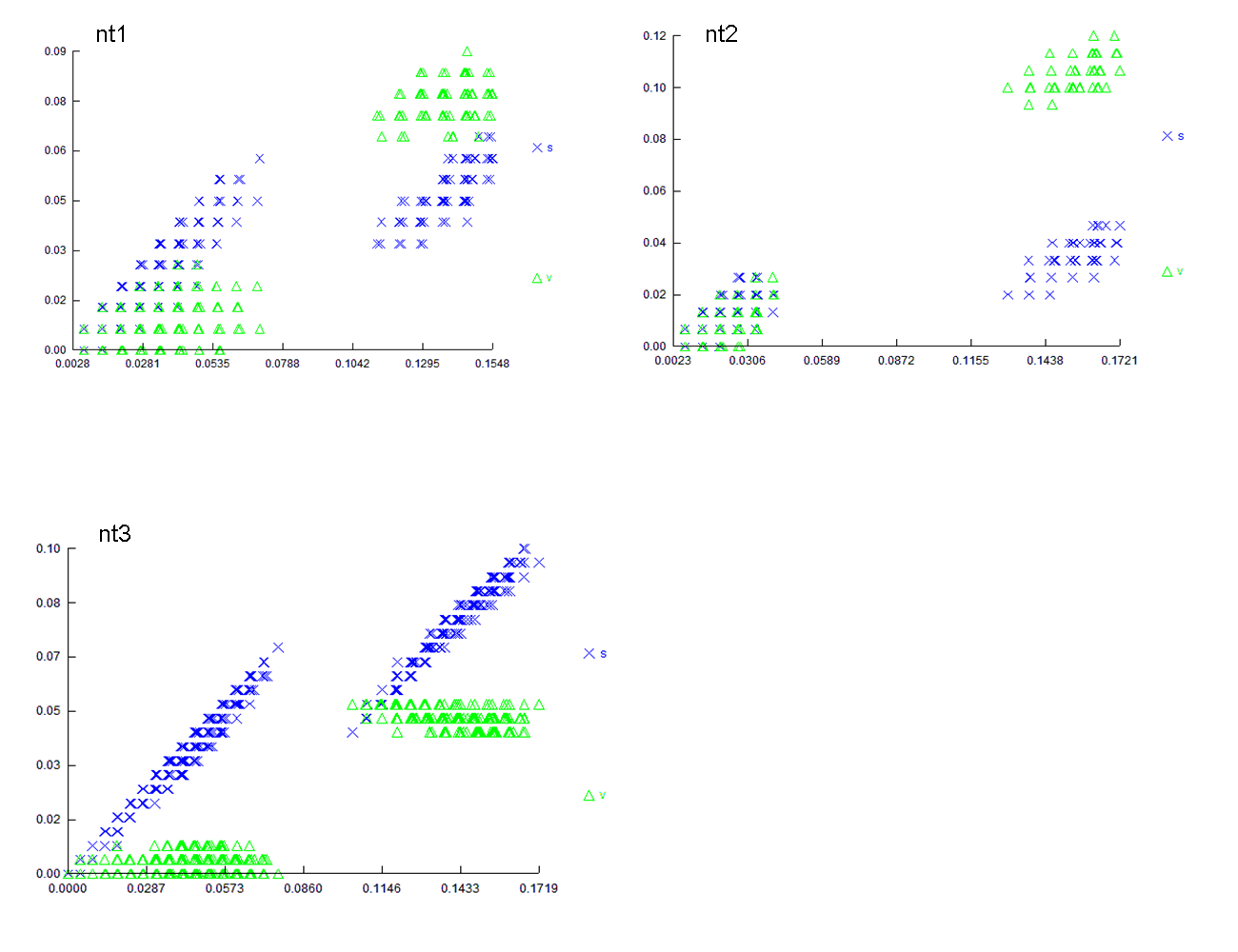

Supplement: Additional file 1 — Numbers of transitions (X and s) and transversions (Δ and v) at each codon position plotted against Tamura-Nei (Tamura and Nei, 1993) genetic distances for the mitochondrial COI gene. The steeper slope of the transitions in nt3 suggests no substitution saturation and is evidence for a constant rate of evolution. [file 1756-3305-6-47-S1.tiff]
